# RETRACTION: The Role and Molecular Mechanism of Trop2 Induced Epithelial–Mesenchymal Transition Through Mediated β‐Catenin in Gastric Cancer

**DOI:** 10.1002/cam4.70629

**Published:** 2025-02-23

**RETRACTION**: W. Zhao, L. Jia, X. kuai, Q. Tang, X. Huang, T. Yang, Z. Qiu, J. Zhu, J. Huang, W. Huang, and Z. Feng, “The Role and Molecular Mechanism of Trop2 Induced Epithelial‐Mesenchymal Transition Through Mediated β‐catenin in Gastric Cancer,” *Cancer Medicine* 8, no. 3 (2019): 1135–1147, https://doi.org/10.1002/cam4.1934.

The above article, published online on 11 January 2019 in Wiley Online Library (wileyonlinelibrary.com), has been retracted by agreement between the journal Editor‐in‐Chief, Stephen Tait, and John Wiley & Sons Ltd. The retraction has been agreed due to concerns raised by third parties. Further investigation by the publisher has unveiled duplication of image elements and/or post‐acquisition image manipulation affecting Figures 2B, 3I and S1A. Furthermore, several cell lines used in this study have been reported as problematic (BGC‐823 [1, 2], MGC‐803 [3, 4], MKN‐28 [5] and SGC‐7901 [1, 2]). Accordingly, the article must be retracted as its conclusions are considered invalid by the editors. The authors were informed of the decision of retraction but were unavailable for a final confirmation.
